# Supplementary material for: MIS-Net: A deep learning-based multi-class segmentation model for CT images
Source: PLoS One. 2024 Mar 13;19(3):e0299970. doi: 10.1371/journal.pone.0299970 (PMC10936843; doi:10.1371/journal.pone.0299970)
Supplement: Attachment — Submitted filename: Response to Reviewers.docx [file pone.0299970.s001.docx]

Dear Dr. Priyadarsan Parida,

On behalf of all the contributing authors, I would like to express our sincere appreciations of your letter and reviewers’ constructive comments concerning our article entitled “MIS-Net:a deep learning-based multi-class segmentation model for CT images” (Manuscript No.: PONE-D-23-37619).

We were pleased to know that our work was rated as potentially acceptable for publication in *PLOS ONE*, subject to adequate revision. We thank the editors and reviewers for the time and effort that they have put into reviewing the previous version of the manuscript. Their suggestions have enabled us to improve our work. Based on the instructions provided in your letter, we uploaded the file of the revised manuscript. Accordingly, we have uploaded a copy of the original manuscript with all the changes highlighted by using the track changes mode in Latex.

These comments are all valuable and helpful for improving our article. According to editors comments, we have made extensive modifications to our manuscript and supplemented extra experiments to make our results convincing.

Point-by-point responses to nice editors are listed below this letter.

The editor's comments are laid out below in *italicized font*, and specific concerns have been numbered. Our response is provided in normal font.

1. *Reduce the Introduction Part. Only discuss about the deep learning based approaches.*

We have eliminated the traditional methods from the Introduction Part, opting to focus solely on the discussion of deep learning methods（page1 and 2）.

2. *Provide a proper network architecture indicating the type of filter and the layer in details.*

We have added the suggested content to the manuscript on Model Part. In the encoding module and decoding module of the Model section, we've incorporated details regarding the filters and layers. Additionally, visual representations of these details are now provided in Figures 2 and 3 on pages 5 and 6, respectively

3. *Compare the network with other performance parameters such as Accuracy, Sensitivity, Specificity etc.*

We agree that this is a potential limitation of the study. We have supplemented the Evaluation Metrics section by introducing the metrics Accuracy, Sensitivity, and Specificity along with their respective formulas. Additionally, in the Comparison Experiments section, we have included experimental results for these three metrics in Tables 1, 2, and 3 on pages 12, 13, and 14, respectively.

4. *Compare your network with other recent state of art approaches to prove the superiority of your proposed approach.*

In response to the editors' suggestion, we have included a comparative experiment with the Auto-model proposed in 2022 in the Comparison Experiments section. The results of this experiment have been incorporated into Tables 1 and 2 (on pages 12 and 13) and Figures 7, 8, 11, and 12 (on pages 12 and 15).

We made our best efforts to enhance the manuscript and implemented various changes. These modifications do not affect the content or structure of the paper. In the revised version, deletions in our manuscript are highlighted in red text, and additions are marked in blue throughout the document.

We appreciate for Editors’ warm work earnestly, and hope that the correction will meet with approval.

Once again, thank you very much for your comments and suggestions.

Yours sincerely,

Dr. Changying Wang

[wcing@qdu.edu.cn](mailto:wcing@qdu.edu.cn)

December 6, 2023

QingDao University
