# Supplementary material for: MIS-Net: A deep learning-based multi-class segmentation model for CT images
Source: PLoS One. 2024 Mar 13;19(3):e0299970. doi: 10.1371/journal.pone.0299970 (PMC10936843; doi:10.1371/journal.pone.0299970)
Supplement: Attachment — Submitted filename: plos1_review.docx [file pone.0299970.s002.docx]

Although the authors have done really nice job in incorporating suggested review comments but still the incorporation of the following points will help in enhancing its quality:

1. Although you have added one 2022 technique but still, I think incorporating comparison with more techniques of 2022 and 2023 (at least 3-4) will add value to your manuscript. So, please try to incorporate it. It will give a clear validation to the readers about the significance of your proposed technique.
2. There is no information given regarding the Future work for the proposed methodology. Please include it.
3. Please clearly depict the USP and significance of your research work for the chosen research problem.
4. In the References, please give more focus on new methodologies of 2022 and 2023.
