# Supplementary material for: MIS-Net: A deep learning-based multi-class segmentation model for CT images
Source: PLoS One. 2024 Mar 13;19(3):e0299970. doi: 10.1371/journal.pone.0299970 (PMC10936843; doi:10.1371/journal.pone.0299970)
Supplement: Attachment — Submitted filename: Response to Reviewers.docx [file pone.0299970.s003.docx]

Dear Editors and Reviewers,

On behalf of all the contributing authors, I would like to express our sincere appreciations of your letter and reviewers’ constructive comments concerning our article entitled “MIS-Net:a deep learning-based multi-class segmentation model for CT images” (Manuscript No.: PONE-D-23-37619).

We were pleased to know that our work was rated as potentially acceptable for publication in *PLOS ONE*, subject to adequate revision. We thank the editors and reviewers for the time and effort that they have put into reviewing the previous version of the manuscript. Their suggestions have enabled us to improve our work. Based on the instructions provided in your letter, we uploaded the file of the revised manuscript. Accordingly, we have uploaded a copy of the original manuscript with all the changes highlighted by using the track changes mode in Latex.

These comments are all valuable and helpful for improving our article. According to editors comments, we have made extensive modifications to our manuscript and supplemented extra experiments to make our results convincing.

Point-by-point responses to nice editors are listed below this letter.

The reviewers’ comments are laid out below in *italicized font*, and specific concerns have been numbered. Our response is provided in **bold** font.

Reviewer #1:

*1. Although you have added one 2022 technique but still, I think incorporating comparison with more techniques of 2022 and 2023 (at least 3-4) will add value to your manuscript. So, please try to incorporate it. It will give a clear validation to the readers about the significance of your proposed technique.*

**We have added three comparative experiments for the technologies proposed in 2022-2023 in the Comparison Experiments section of the article, which are MLP-Mixer, DALU-Net, and Modified 3D U-Net, in page 11, line 356, and page 12, lines 375-236. The comparison results are shown in Figs 7,8 and Tables 1,2 in pages 12-13.**

*2. There is no information given regarding the Future work for the proposed methodology. Please include it.*

**We are very sorry for our negligence of future work for the proposed methodology, we have added future work to the Conclusion and Discussion section, which is on page 16, line 437 of the article.**

*3. Please clearly depict the USP and significance of your research work for the chosen research problem.*

**We are very sorry for our incorrect writing USP and significance of my research work, we have corrected it in the Introduction section in page 2, lines 61-76 and added the significance of the work in the Conclusion and Discussion section in page 16, lines 426-430.**

*4. In the References, please give more focus on new methodologies of 2022 and 2023.*

**We added four 2022-2023 references to be used as comparative experiments and literature references, Refs. 27, 34, 35, 36**

Reviewer #2: 
*1.Clinical applications of proposed MIS-Net and how?*

**We have added the clinical applications of MIS-Net in Conclusion and Discussion section in page 16, lines 421-425.**

*2.Authors missed some existing prominent works of multi-class segmentation and should discuss them critically in introduction section 10.1109/ACCESS.2019.2896961; 10.1007/978-981-13-1702-6_53; 10.3390/bioengineering9110709.*

**We sincerely appreciate the valuable comments. We have checked the literature carefully and added more references on 10.1109/ACCESS.2019.2896961 and 10.1007/978-981-13-1702-6_53 into the Introduction section in the revised manuscript in page 2, lines 47-55. We have added reference on 10.3390/bioengineering9110709 into the Related Work section in page 3, lines 98-100.**

*3.“Effectively reduces the computational load during network training, and accelerates model convergence’, what is experimental justification of this statement?*

**We added a graph comparing the training set loss curves for the first 100 epochs of VNet and MIS-Net training as a demonstration of the above problem in page 15, Fig 11.**

*4.Why did it need “Data Enhancement”, authors already used two datasets.*

**Although this article conducted model training in two data sets, the number of data samples contained in each data set is not large, of which covid-19 only includes 20 samples and lits17 only has 200 samples.**

**So this paper adopts the data augmentation method of rotation and flipping to solve the data shortage problem by creating more data from the available data.**

*5.Authors did not mention implementation challenges.*

**Considering the reviewer’s suggestion, we have added implementation challenges to the Conclusion and Discussion section in pages 16-17, lines 440-444.**

We made our best efforts to enhance the manuscript and implemented various changes. In the revised version, deletions in our manuscript are highlighted in red text, and additions are marked in blue throughout the document.

We sincerely hope that our revised manuscript meets the standards for publication in PLOS ONE. If further revisions are necessary, please feel free to contact me at [wcing@qdu.edu.cn](mailto:wcing@qdu.edu.cn).

We appreciate for editors’ and reviewers’ warm work earnestly, and hope that the correction will meet with approval.

Once again, thank you very much for your comments and suggestions.

Best regards,

Dr. Changying Wang

[wcing@qdu.edu.cn](mailto:wcing@qdu.edu.cn)

January 12, 2024

QingDao University
