# Supplementary material for: MIS-Net: A deep learning-based multi-class segmentation model for CT images
Source: PLoS One. 2024 Mar 13;19(3):e0299970. doi: 10.1371/journal.pone.0299970 (PMC10936843; doi:10.1371/journal.pone.0299970)
Supplement: Attachment — Submitted filename: acceptance_review.docx [file pone.0299970.s004.docx]

Dear authors, thank you for addressing all my concerns. No further suggestions from me.
