# Supplementary material for: MIS-Net: A deep learning-based multi-class segmentation model for CT images
Source: PLoS One. 2024 Mar 13;19(3):e0299970. doi: 10.1371/journal.pone.0299970 (PMC10936843; doi:10.1371/journal.pone.0299970)
Supplement: Attachment — Submitted filename: Response to Reviewers.docx [file pone.0299970.s005.docx]

Dear Editors and Reviewers,

On behalf of all the contributing authors, I would like to express our sincere appreciations of your letter and reviewers’ constructive comments concerning our article entitled “MIS-Net:a deep learning-based multi-class segmentation model for CT images” (Manuscript No.: PONE-D-23-37619).

We were pleased to know that our work was rated as potentially acceptable for publication in *PLOS ONE*, subject to adequate revision. We thank the editors and reviewers for the time and effort that they have put into reviewing the previous version of the manuscript. Their suggestions have enabled us to improve our work. Based on the instructions provided in your letter, we uploaded the file of the revised manuscript. Accordingly, we have uploaded a copy of the original manuscript with all the changes highlighted by using the track changes mode in Latex.

These comments are all valuable and helpful for improving our article. According to editors comments, we have made extensive modifications to our manuscript and supplemented extra experiments to make our results convincing.

Point-by-point responses to nice editors are listed below this letter.

The reviewers’ comments are laid out below in *italicized font*, and specific concerns have been numbered. Our response is provided in **bold** font.

Reviewer #1:

**It is a great honor to receive your approval of this work, and thank you for your comments to improve the quality of the article!**

Reviewer #2: 
*1. Authors should give detailed description of Figure 1.*

**We think this is an excellent suggestion. We have explained the change made, including the exact location where the change can be found in the revised manuscript in page 4, lines 147-151.**

*2. Authors should describe what phenomenon in Figure 11.*

**Thank you for your suggestion. As suggested by reviewer, we have added the suggested content to the manuscript on pages 14-15, lines 414-418.**

*3. An introduction is an important road map for the paper that should be consist of an opening hook to catch the researcher's attention. To make soundness of your study should include these latest related works*

**We sincerely appreciate the valuable comments. We have checked the literature carefully and added more references on 10.3389/fbioe.2020.00897, 10.1016/j.icte.2024.01.002 and 10.1109/JBHI.2023.3298708 into the Introduction section in the revised manuscript in page 2, lines 57-64. We have added reference on 10.3389/fneur.2023.1148846 into the Introduction section in page 1, lines 10-13.**

*4. Authors should be carefully check all references, volume, issue, page number etc.*

**We are very grateful to this suggestion, based on which we carefully checked all references.**

We made our best efforts to enhance the manuscript and implemented various changes. In the revised version, deletions in our manuscript are highlighted in red text, and additions are marked in blue throughout the document.

We sincerely hope that our revised manuscript meets the standards for publication in PLOS ONE. If further revisions are necessary, please feel free to contact me at [wcing@qdu.edu.cn](mailto:wcing@qdu.edu.cn).

We appreciate for editors’ and reviewers’ warm work earnestly, and hope that the correction will meet with approval.

Once again, thank you very much for your comments and suggestions.

Best regards,

Dr. Changying Wang

[wcing@qdu.edu.cn](mailto:wcing@qdu.edu.cn)

February 7, 2024

QingDao University
